# Fermentation Improves the Antioxidant Activity, Flavor Components and Metabolic Characteristics of Coffee Pulp Beverage

**DOI:** 10.3390/microorganisms14081839

**Published:** 2026-08-19

**Authors:** Runhua Gong, Weixin Sun, Tao Pan, Li Zhuang, Shichao Peng, Jianyong Wang, Mingduo Yang, Jingjing Bai, Lili Zhao

**Affiliations:** 1 College of Economics and Management, Pu’er University, Pu’er 665000, China; gongrunhua@peu.edu.cn (R.G.); yangmingduo5663@163.com (M.Y.); 2 Center of Development and Planning, Pu’er University, Pu’er 665000, China; sunweixin@peu.edu.cn (W.S.);; 3 College of Tea and Coffee, Pu’er University, Pu’er 665000, China

**Keywords:** coffee pulp beverage, fermentation, volatile compounds, antioxidant activity, metabolites

## Abstract

This study aimed to evaluate the impact of four strains, *Bifidobacterium longum* BL11, *Streptococcus thermophilus* LB42, *Lacticaseibacillus casei* LC15, and *Lactiplantibacillus plantarum* XJ25, on the antioxidant activity, flavor components and metabolic characteristics of fermented coffee pulp beverage (CPB). Results indicated that the antioxidant activities were remarkably enhanced in the fermented CPB samples (*p* < 0.05). A total of 103 volatile compounds (VOCs) were detected by HS-SPME-GC-MS and GC-IMS, with 37 types of VOCs newly generated after fermentation, including esters, alcohols and ketones, thereby enhancing the flavor quality of CPB. In addition, a total of 334 differential non-volatile metabolites covering 20 subclasses were obtained in all CPB samples, such as D-tagatose, glyceraldehyde, isorhynchophylline and pyruvic acid, which showed remarkably increased levels after fermentation (*p* < 0.05). Organic acids, amino acids and derivatives were the two major classes of differential metabolites. The CPB samples had a different flavor profile after fermentation, while the group of *Lactiplantibacillus plantarum* showed the highest overall acceptability. These findings provide a systematic comparison of single-strain fermentation effects on VOCs and non-volatile metabolites in CPB samples, promoting its high-value utilization in innovative food applications.

## 1. Introduction

Coffee has become one of the most significant non-alcoholic beverages worldwide, valued for its sensory complexity, potential health benefits and economic importance [1,2]. However, the processing of coffee produces large volumes of by-products, such as mucilage, pulp and parchment. Among these, coffee pulp (CP) is the most typical by-product, accounting for approximately 40–50% of the total weight of fresh coffee fruit [3,4]. CP exhibits a high nutrient content and is described to encompass 45–89% carbohydrates, 13–21% fiber, 6–10% minerals, 4–12% proteins and 1–2% lipids, depending on the location, variety and planting methods of the coffee [5]. Moreover, CP is rich in various bioactive compounds such as polyphenolic compounds, gallic acid, chlorogenic acid, hydroxycinnamic acid, rutin, anthocyanins, caffeine and flavonoids [6,7,8,9,10], which may contribute to reducing the risks of chronic diseases such as cancer and cardiovascular disease [11,12]. It has been reported that over 9 million tons of CP are produced in annual coffee production yields, accounting for 62.6% of the total coffee by-products [13]. Regrettably, despite its abundance and high nutritional potential, the utilization remains limited at present with a large amount of CP discarded untreated during traditional coffee processing, leading to significant resource waste and environmental impacts [14].

Notably, CP has been officially designated as a “new food” by the European Union [15]. The addition of CP has remarkably increased the bioactive capacity, nutritional value, flavor properties and sensory in the production of biscuits [16]. The fermentation of co-culture with indigenous *Saccharomyces cerevisiae* and *non-Saccharomyces* in CP wine could enhance its physicochemical, sensory and aromatic characteristics [17]. Additionally, the fermentation of co-culture with *Saccharomyces boulardii* and *Lactiplantibacillus plantarum* in extracted coffee cherry pulp could enhance its sensory and antioxidant properties [18]. Moreover, CP can be processed into CP tea; compared to coffee, CP tea possesses floral, sweet and tea-like characteristics, combining the flavors of tea and fruit [19]. Therefore, in light of the current underdeveloped state of CP deep processing, the fermented coffee pulp beverage (CPB) industry harbors considerable untapped potential.

Fermentation, a biotechnological process, is widely known for improving nutritional properties and transforming raw materials into food products that are more easily used and absorbed by the human body through microbial metabolism [16,20]. With the increasing consumer popularity for fruit beverages fermented by specific strains, sales are expected to reach 65.69 billion USD by 2029 [21,22]. Additionally, fruit beverages are abundant in various nutrients and serve as perfect materials for the growth and fermentation of strains. Most studies have indicated that fermentation can influence the flavor of plant-based beverages, for example, the production of some flavors, such as acids, ketones, alcohols, aldehydes and esters [23,24,25]. Moreover, fermentation is also able to influence the levels of antioxidant activity [26]. For example, the contents of flavonoid and total phenol, as well as antioxidant activity, have significantly increased in citrus juice fermented by *Lacticaseibacillus fermentum* 252 [27]. Some strains were proven to enhance the metabolic capacities and flavor of fruit beverages after fermentation [28], but not all the strains were suitable for CPB fermentation to improve its quality. Therefore, it was necessary to select the strains specifically suitable for CPB fermentation for precise regulation of the flavor and function. CPB is a novel fermented beverage with a profitable taste. Due to the contents of flavor compounds inherent in the CP being relatively low, the main source of flavor is attributed to the fermentation in CPB. Therefore, the vital role of strains in forming the characteristic flavor of CPB was emphasized. However, studies of fermentation from CPB and its effects on physicochemical parameters and metabolites are limited. Additionally, the probiotic attributes of beverages fermented with different strains could vary significantly [29]. Therefore, studying the fermentation of CPB using different strains is an important direction for future scientific research.

This study hypothesized that fermentation of CPB using different strains would result in distinct differences in bioactivities, flavor, and metabolite characteristics, depending on the strains employed. To address these gaps, this study employs quantitative analysis of physicochemical characteristics, along with metabolomics, to comprehensively explore the impact of *Bifidobacterium longum* BL11, *Lacticaseibacillus casei* LC15, *Streptococcus thermophilus* LB42 and *Lactiplantibacillus plantarum* XJ25 on the metabolic components in CPB samples. It is important to note that fermentation outcomes are inherently strain-dependent. This work aims to provide a theoretical foundation for enhancing CPB flavor and functional quality through targeted strain selection.

## 2. Materials and Methods

### 2.1. Materials

Arabica coffee, which was harvested at commercial maturity on November 2024, Pu’er City, Yunnan Province, China, was used. The sampling work was conducted by the College of Tea and Coffee, Pu’er University. *L*. *plantarum* XJ25 was obtained from the College of Enology, Northwest A&F University (Yangling, China). *L*. *casei* LC15, *B*. *longum* BL11 and *S*. *thermophilus* LB42 were purchased from Xi’an Mixier Biotechnology Co., Ltd. (Xi’an, China). All strains were cultured using the MRS medium. 1,1-diphenyl-2-trinitrophenylhydrazine (DPPH) and 2,2′-hydrazine-bis (3-ethylbenzothiazolin-6-sulfonic acid) diamine salt (ABTS) were purchased from Aladdin. Folin-phenol (Beijing Solaibao Technology Co., Ltd., Beijing, China), gallic acid (Shanghai Zhanyun Chemical Co., Ltd., Shanghai, China), and all other reagents are of analytical grade. Sodium nitrite, ferrous sulfate, aluminum nitrate, hydrogen peroxide, trichloroacetic acid, phosphoric acid, ferric chloride (FeCl_3_), glutamate, potassium ferrocyanide (K_3_[Fe(CN)_6_]), n-butanol, and anhydrous ethanol were all purchased from Shanghai McLean Biochemical Technology Co., Ltd. (Shanghai, China) and Tianjin Fuyu Fine Chemical Co., Ltd. (Tianjin, China).

### 2.2. Fermentation Design

The four strains were prepared using the method of Zhao et al. [30]. Fresh CP was thoroughly cleaned twice with distilled water and dried in a hot-air oven at 80 °C for 12 h. Then, the CP and heated water were mixed in a 1:100 ratio and commercial sucrose was added at a concentration of 100 g/L. The mixture was autoclaved at 115 °C for 20 min to prevent spontaneous fermentation. Each strain was inoculated at 0.02% (*w*/*w*) into separate 500 mL sealed glass bottles containing 300 mL of CP soup. Fermentation proceeded at 37 °C under static (semi-anaerobic) conditions for 48 h. During fermentation, 0 h, 6 h, 12 h, 24 h, 36 h, and 48 h CPB samples were collected to measure the physicochemical and microbiological parameters; the 48 h fermentation samples were collected for the detection of other indicators. All samplings were taken from the same batch. This experiment was repeated three times.

The experiment involved five treatment groups: Y (sterilized, unfermented control); YC (*B. longum* BL11); YG (*L. casei* LC15); YS (*S. thermophilus* LB42); and YX (*L. plantarum* XJ25).

### 2.3. Measurement of Physicochemical Properties

The pH of CPB samples was detected directly using a pH meter (PB-10, Sartorius Instrument Co., Ltd., Göttingen, Germany). Total acidity was determined via acid–base titration and expressed as g/L (citric acid equivalent). The reducing sugar content was detected using the method previously published [23]. Soluble solids content (°Brix) was measured using a digital refractometer.

### 2.4. Measurement of Viable Counts

Prior to quantifying cells, CPB samples were diluted in sterile 0.85% (*w*/*v*) NaCl solutions. A 100 μL aliquot of each dilution was plated onto MRS plates and incubated at 37 °C for 30 h [30].

### 2.5. Analysis of Antioxidant Activity

#### 2.5.1. Total Flavonoid and Total Phenolic

The total flavonoid content was assessed using the method previously published by Zhou et al. [31]. Total phenolic content was assessed using the method of Yue et al. [26]. Briefly, 0.5 mL of CPB sample was taken and mixed with 1 mL of 0.1 mol/L phenol solution and 1 mL of 0.2 g/mL Na_2_CO_3_ solution, and then the absorbance was measured at 765 nm using an OLB-V1000G spectrophotometer (High Precision Biological Diagnosis and Analysis Industry Technology Research Institute Co., Ltd., Jinan, Shandong, China).

#### 2.5.2. DPPH and ABTS Radical Scavenging Rates

The radical scavenging rates of DPPH and ABTS were assessed using the method previously reported by Song et al. [32].

#### 2.5.3. Ferric Reducing Antioxidant Power (FRAP)

FRAP was measured using the method previously published by Ding et al. [33] with little modifications. The concentration of 0.2 mol/L phosphate buffer (pH 6.6) was prepared. K_3_[Fe(CN)_6_] solution (1%) and trichloroacetic acid solution (10%) were also prepared for later use. Similarly, 0.1 g FeCl_3_ was dissolved in distilled water to obtain a 0.1% FeCl_3_ solution. Then, 1.0 mL each of CPB sample was taken and diluted with distilled water to prepare 5 sample solutions with different concentrations (0.2 mg/mL, 0.4 mg/mL, 0.8 mg/mL, 1 mg/mL, and 2 mg/mL). Next, 2.5 mL of each CPB solution was taken, mixed with 2.5 mL each of phosphate buffer and K_3_[Fe(CN)_6_] solutions, and then incubated at 55 °C for 30 min. After the mixtures were cooled, they were mixed with 2.5 mL trichloroacetic acid and centrifuged at 10,000 *g* for 10 min. Finally, 2.5 mL of sample supernatant was taken, and 2.5 mL of distilled water and 0.5 mL of FeCl_3_ solution were added. The absorbance was detected at 700 nm (A). The positive control experiment was carried out with glutamate (CK), and the blank control experiment was not added with FeCl_3_ solution (A_0_).

Restoring power = (A − A_0_)

### 2.6. Determination of Volatile Compounds (VOCs)

#### 2.6.1. Electronic Nose (E-Nose)

The volatile flavor substances in CPB samples were detected using an E-Nose (cNose, Shanghai Baosheng Industrial Development Co., Ltd., Shanghai, China). The E-Nose system consists of 14 different sensors, each sensitive to specific volatile compounds, and is used to measure the overall flavor of CPB [34]. The main responsive substances corresponding to the sensor are shown in Table S1. An amount of 20 mL of the sample stood in a constant-temperature box at 37 °C for 30 min before measurement. The measurement conditions were as follows: 400 mL/min injection flow rate, 120 s sensor self-cleaning time and 60 s detection time.

#### 2.6.2. Headspace Solid-Phase Microextraction-Gas Chromatography-Mass Spectrometry (HS-SPME-GC-MS)

The VOCs in CPB were extracted using HS-SPME and analyzed by GC-MS according to the method previously reported [35]. Samples (5 mL) were transferred to a 20 mL headspace glass vial (ZZ-SPME-Sab, Qingdao Zhenzheng Analytical Instrument Co., Ltd., China, Qingdao, China) containing 1.3 g NaCl; then, 10 μL of 2-octanol (B24648, Shanghai Yuanye Biotechnology Co., Ltd., China, Shanghai, China) was added as an internal standard at a concentration of 2 g/L. Subsequently, each vial was set in a metal bath at 60 °C for 30 min; then, a 120 μm CAR/PDMS/DVB fiber (SPME-06s, Qingdao Zhenzheng Analytical Instrument Co., Ltd., China) was exposed to the headspace of the sample for 30 min at 60 °C. The analysis was performed by GC-MS (7890-5977B, Agilent, Santa Clara, CA, USA) equipped with a 19091 S-433UI HP-5MS chromatographic column (10 m × 0.25 mm × 0.25 μm, Agilent, USA).

#### 2.6.3. Gas Chromatography-Ion Mobility Spectrometry (GC-IMS)

On the other hand, the VOCs of CPB samples were detected using a GC-IMS instrument (FlavourSpec^®^, GAS, Dortmund, Germany) equipped with an MXT-WAX capillary column (30 m × 0.53 mm, 1.0 μm, Restek, Bellefonte, PA, USA). According to the previous methodology with little modifications [28], 300 μL of CPB sample was added to a headspace vial, then incubated at 500 rpm and 60 °C for 15 min. The programming of gas chromatography was set as follows: initial flow rate of 2 mL/min for 0–2 min, followed by increases to 10 mL/min during 8 min and subsequently to 100 mL/min during 10 min, with final maintenance for 20 min. IMS conditions: the flowing gas was nitrogen (N2, 99.999% purity), the E1 flowing gas flow rate was 75 mL/min, and the IMS temperature was 45 °C. The VOCs were analyzed qualitatively using GC database and IMS database. Furthermore, the fingerprint maps and 2D difference maps of VOCs were created using analysis tools with the GC-IMS system (VOCal v1.0.3, GAS, Dortmund, Germany).

### 2.7. Ultra-Performance Liquid Chromatography Dual Mass Spectrometry (UPLC-MS/MS)

The non-volatile metabolites were detected as previously described with minor modifications [32]. A 100 μL CPB sample was transferred into a 1.5 mL centrifuge tube; 400 μL of 70% methanol internal standard was added. Next, the sample was shaken for 15 min and centrifuged for 3 min (12,000 r/min, 4 °C). Subsequently, the supernatant was collected and filtered into the injection vial for further analysis. The analysis was performed using a coupled system consisting of a UPLC (Vanquish, Thermo Scientific, Waltham, MA, USA) equipped with an ACQUITY UPLC HSS T3 column (1.8 μm, 2.1 mm × 100 mm, Waters, Milford, MA, USA) and a Q Exactive HF-X high-resolution MS (Thermo Scientific, Waltham, MA, USA).

### 2.8. Sensory Evaluation of CPB

The sensory evaluation followed the method of Moukamnerd et al. [36] with some modifications. Twelve sensory evaluators (6 men and 6 women) were trained to describe and identify the sensory quality of CPB samples. Evaluators rated CPB samples on aroma, appearance, taste and overall acceptability.

### 2.9. Statistical Analysis

The data were the average values of three replicates. One-way ANOVA followed by Tukey–Kramer post hoc test was employed to test the significant differences (*p* < 0.05). Data processing and graphing were performed using Simca.14.1, Origin 2019, GraphPad Prism 9.0.0, and SPSS 26.

## 3. Results and Discussion

### 3.1. Dynamic Changes in Physicochemical and Microbiological Parameters

**Figure 1:**
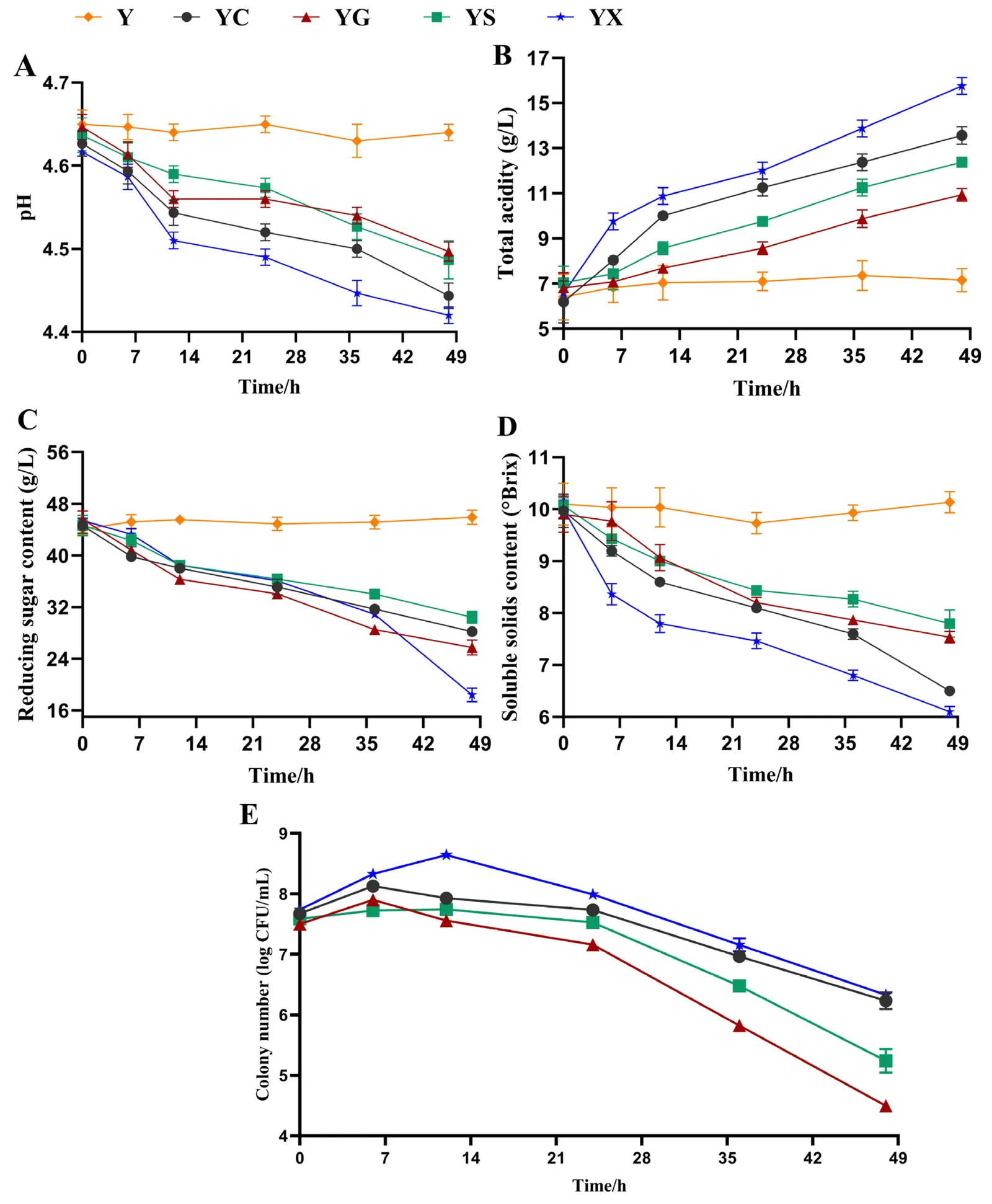
Changes in physicochemical and microbiological parameters during CPB fermentation. (**A**) pH. (**B**) Total acidity (g/L, citric acid equivalent). (**C**) Reducing sugar content. (**D**) Soluble solids content (°Brix). (**E**) Viable bacterial counts. Y: sterilized, unfermented control; YC: *B. longum* BL11; YG: *L. casei* LC15; YS: *S. thermophilus* LB42; YX: *L. plantarum* XJ25.

Changes in pH and total acidity are displayed in Figure 1A,B. The pH values reduced linearly during the whole fermentation in all groups (*p* < 0.05), mainly due to the production of some organic acids by fermentation. After 48 h fermentation, the YX sample showed the most pronounced pH decrease from 4.62 to 4.42, while the YC, YG and YS samples declined to 4.44, 4.50 and 4.49, respectively. The total acidity showed an opposite trend to the pH; the total acidity increased and varied significantly among all CPB samples, corresponding to the utilization of different strains. The total acidity of YG sample fermented by *L. casei* ranged from 6.81 to 10.94 g/L, which was much lower than others.

Reducing sugars act as indispensable nutrients in CPB, providing crucial carbon sources for the growth and metabolism of strains [37]. Changes in reducing sugar and soluble solids content are displayed in Figure 1C,D. As fermentation progressed, the sugar content and soluble solids content decreased significantly during each fermentation treatment, indicating that the strains started utilizing the sugars in CPB. After fermentation, the YX sample had the lowest content of reducing sugars, with a decrease of 59.48%, indicating that the carbon source was effectively utilized by *L. plantarum*. It should be noted that the decrease of 59.48% refers to the endogenous reducing sugars (primarily glucose and fructose from the coffee pulp matrix) rather than the added sucrose. Future studies should employ HPLC-based quantification of individual sugars to more accurately track carbon-source utilization. The soluble solids content of YC, YG, YS, and YX were 6.50%, 7.53%, 7.80%, and 6.10%, respectively. The differences in sugar contents among the four CPB samples may be ascribed to disparities in the strain’s capacity to utilize and transform sugars in the fermentation matrix [38]. In this study, *L. plantarum* XJ25 illustrated the lowest pH and sugar content and the highest viable cell count and total acidity. This is similar to results reported by Vivek et al. [39], who found the same trend of abundant production of organic acids through sugar conversion by fermentation.

The viable cell count serves as a critical determinant of both the functionality and quality of fermented foods [40]. Figure 1E illustrates the dynamics of viable counts for four strains (YC, YG, YS, YX) in fermented CPB. The initial counts were 7.50–7.74 log CFU/mL. The highest counts of YC and YG reached 8.13 ± 0.02 and 7.90 ± 0.02 log CFU/mL after 6 h of fermentation, respectively, and then decreased. In contrast, YS and YX reached 7.74 ± 0.06 and 8.64 ± 0.06 log CFU/mL, respectively, and then declined continuously after 12 h of fermentation. At the fermentation of 48 h, the viable count of YX fermented by *L. plantarum* XJ25 was the highest, which was 6.33 ± 0.07 log CFU/mL, while the total number of colonies of YC, YG, and YS were 6.23 ± 0.01, 4.50 ± 0.08 and 5.24 ± 0.16 log CFU/mL, respectively, which all exceeded 4 log CFU/mL. Compared with those of YC, YG and YS, the viable cell count of YX was greater during the whole fermentation, indicating that *L. plantarum* XJ25 more efficiently utilizes the nutritional substances of CPB to support its growth and fermentation, in agreement with the study of Yue et al. [26], who reported that black sapote juice is a better substrate for the growth of *Lacticaseibacillus*.

### 3.2. Changes in Antioxidant Activities

**Figure 2:**
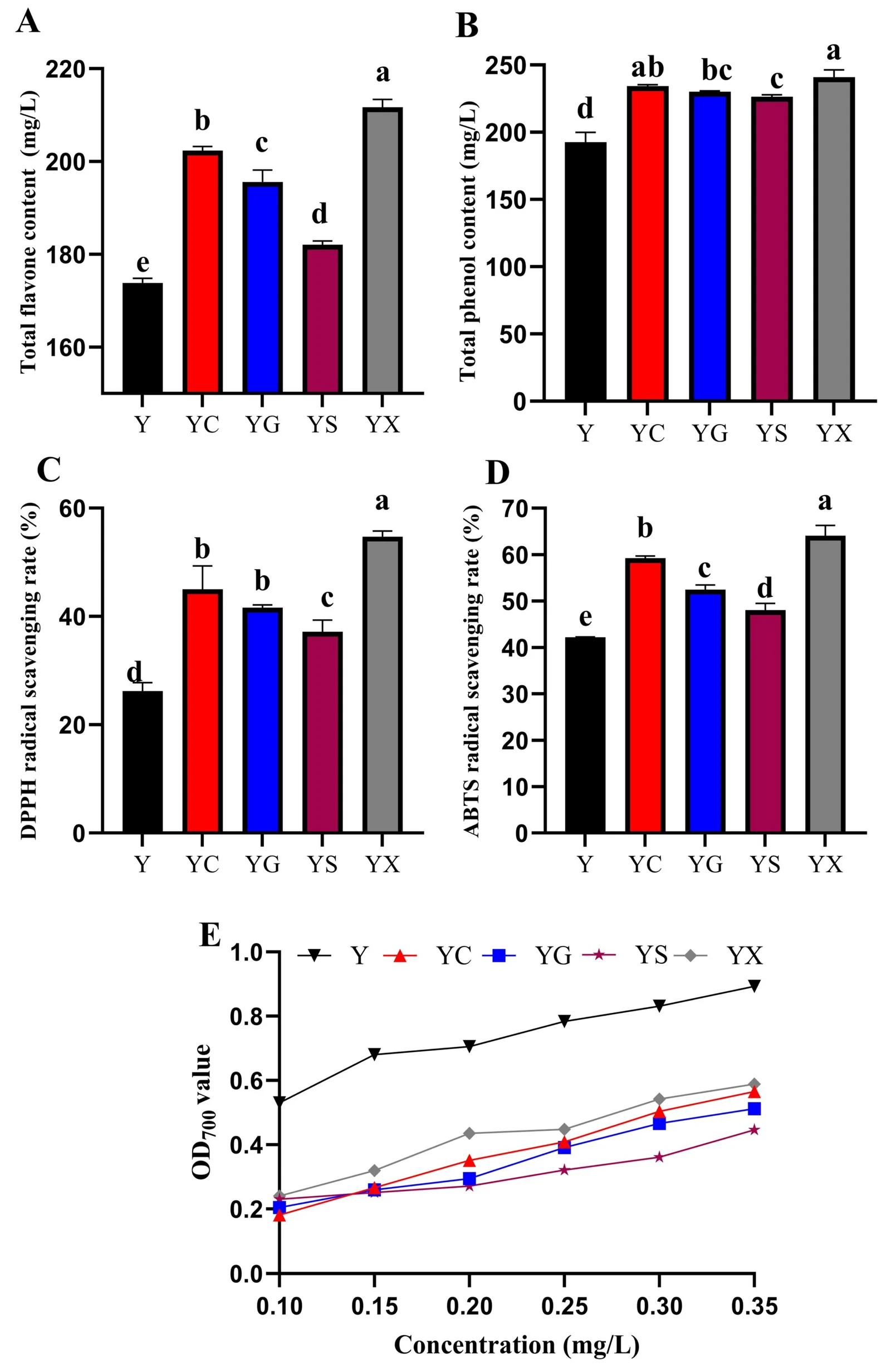
Changes in antioxidant activity of CPB samples. (**A**) Total flavonoids. (**B**) Total phenols. (**C**) DPPH radical scavenging activity. (**D**) ABTS radical scavenging activity. (**E**) Ferric Reducing Antioxidant Power (FRAP). Y: sterilized, unfermented control; YC: *B. longum* BL11; YG: *L. casei* LC15; YS: *S. thermophilus* LB42; YX: *L. plantarum* XJ25. a–e indicate statistical significance determined by one-way ANOVA (*p* < 0.05).

The effect of fermentation on CPB’s antioxidant capacity is displayed in Figure 2A–D. Phenolic and flavonoid compounds have many health benefits, such as anticancer, antioxidant and anti-inflammatory activities [41]. In the original CPB sample, the contents of total flavonoids and phenols were measured at 173.84 ± 0.80 mg/L and 192.63 ± 6.00 mg/L, respectively (Figure 2A,B). Following fermentation, the total flavonoids and phenols increased, with values of 202.37 ± 0.69 mg/L and 234.29 ± 0.91 mg/L for YC, 195.59 ± 2.08 mg/L and 230.13 ± 0.45 mg/L for YG, 182.03 ± 0.69 mg/L and 226.28 ± 1.20 mg/L for YS, and 211.69 ± 1.38 mg/L and 241.03 ± 4.32 mg/L for YX, respectively. This result was consistent with the findings reported by Zhao et al. [42], in which the contents of total flavonoids and phenols in jujube–wolfberry composite juice exhibited a significant increase with fermentation. The increase in total phenol may be attributed to the transformation of complex phenolic compounds to free form by strain depolymerization and hydrolases in fermented CPB samples [43]. The total flavonoids and phenols in the YX sample fermented with *L. plantarum* XJ25 showed a significant increase, much higher than those observed in the Y sample without fermentation, especially the total flavone in CPB with *L. plantarum* XJ25 fermentation which was increased by about 17.88%, and the total phenol was increased by about 20.08% (*p* < 0.05).

The radical scavenging activities of DPPH, ABTS, and FRAP were significantly enhanced in the fermented CPB samples (Figure 2C–E). In the original CPB sample, the radical scavenging activities of DPPH and ABTS were 26.22% and 42.18%, respectively. After fermentation, the values increased to 45.02% and 59.25% for YC, 41.61% and 52.48% for YG, 37.15% and 48.05% for YS, and 54.72% and 64.06% for YX (Figure 2C,D). The observed increase in DPPH radical scavenging activity suggests that fermentation can increase the bioavailability of polyphenolic compounds with proton-donating properties [44]. The YX displayed the highest DPPH and ABTS radical scavenging rates, increasing by 52.08% and 34.15% compared with Y, respectively, highlighting the substantial enhancement of the DPPH and ABTS radical scavenging rates in CPB samples through *L. plantarum* XJ25 fermentation, and the increase in DPPH radical scavenging activity was extraordinarily evident. Additionally, a similar change was observed in the FRAP assay (Figure 2E). As fermentation progressed, the FRAP increased with the concentration of CPB. The FRAP of YS was lower than those of the other four CPB samples, and the highest FRAP was found in YX and YC. Within the tested concentration range, the FRAPs of all five CPB were lower than that of CK (glutamate). Overall, CPB samples fermented with *L. plantarum* XJ25 showed the highest level of antioxidant activity.

### 3.3. Changes in Flavor Characteristics

#### 3.3.1. E-Nose Analysis

**Figure 3:**
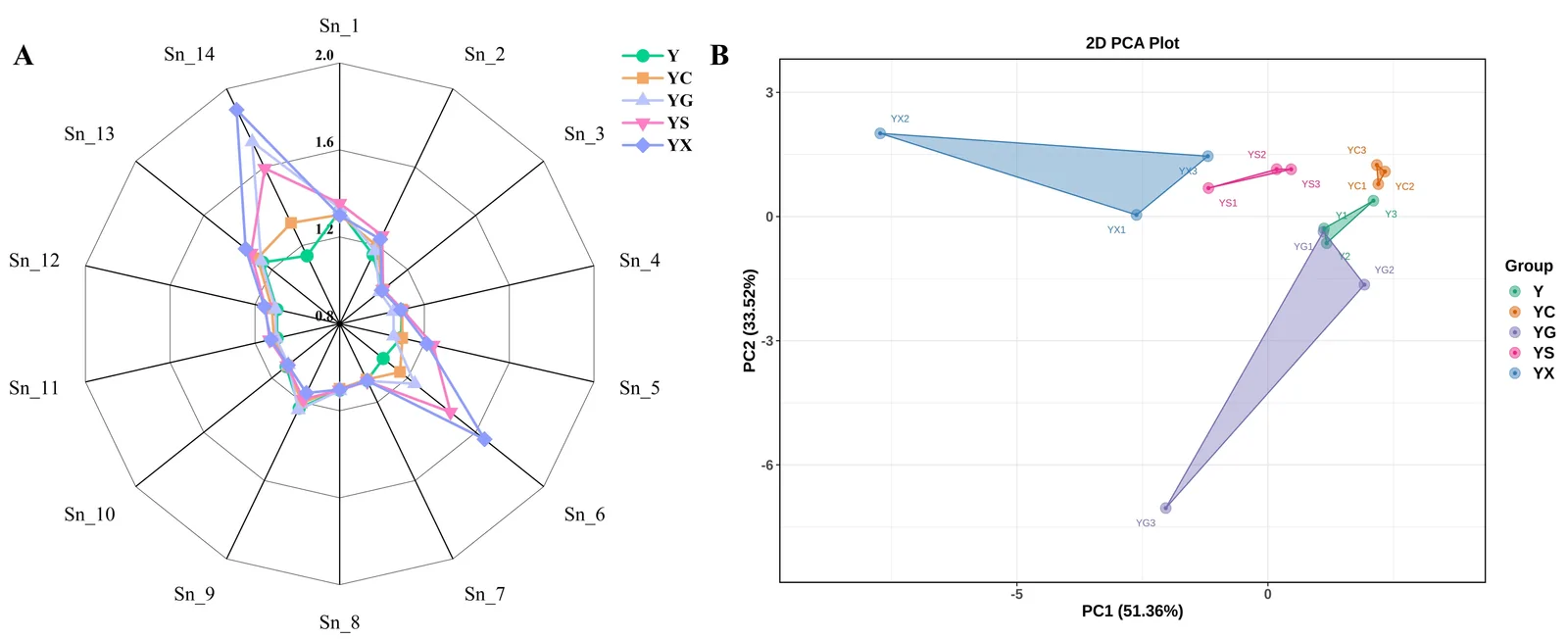
E-nose analysis of CPB samples. (**A**) Radar chart of sensor response values. (**B**) Principal Component Analysis (PCA). Y: sterilized, unfermented control; YC: *B. longum* BL11; YG: *L. casei* LC15; YS: *S. thermophilus* LB42; YX: *L. plantarum* XJ25.

E-Nose technology was used to analyze differences in the flavor of CPBs. As demonstrated in Figure 3A, the response signals of Sn_2, Sn_5, Sn_6, Sn_13 and Sn_14 sensors were much stronger in CPB samples fermented with strains, which indicates that alcohols, aldehydes, nitride, ammonia, benzenes, aldehydes, combustible gases, and aromatic compounds were the main types of volatiles contributing to the differences in CPB samples. In particular, the signal intensities of Sn_6 and Sn_14 in YX were both observed strongest, with YG and YS slightly lower than YX, while the signal intensities of both sensors exhibited the lowest values in Y. Differences from sensors signal (Sn_6 and Sn_14) potentially indicated YX showing stronger aromatic compounds. Principal Component Analysis (PCA) was further used to analyze whether there were differences in the flavor among the 5 CPB samples. The contribution of the first component (PC1) was 51.36%, the second component (PC2) contributed 33.52%, and the total of them was 84.88%, which indicates that the PCA model can well reflect the overall information in differentiating fermented CPBs, and most data obtained from E-nose sensors can be explained by PC1 and PC2 (Figure 3B). Specifically, the Y, YC and YG samples clustered together and were distant from the YS and YX, indicating that the volatile odors of the samples in the fermentation had unique characteristics.

#### 3.3.2. GC-MS Analysis

**Figure 4:**
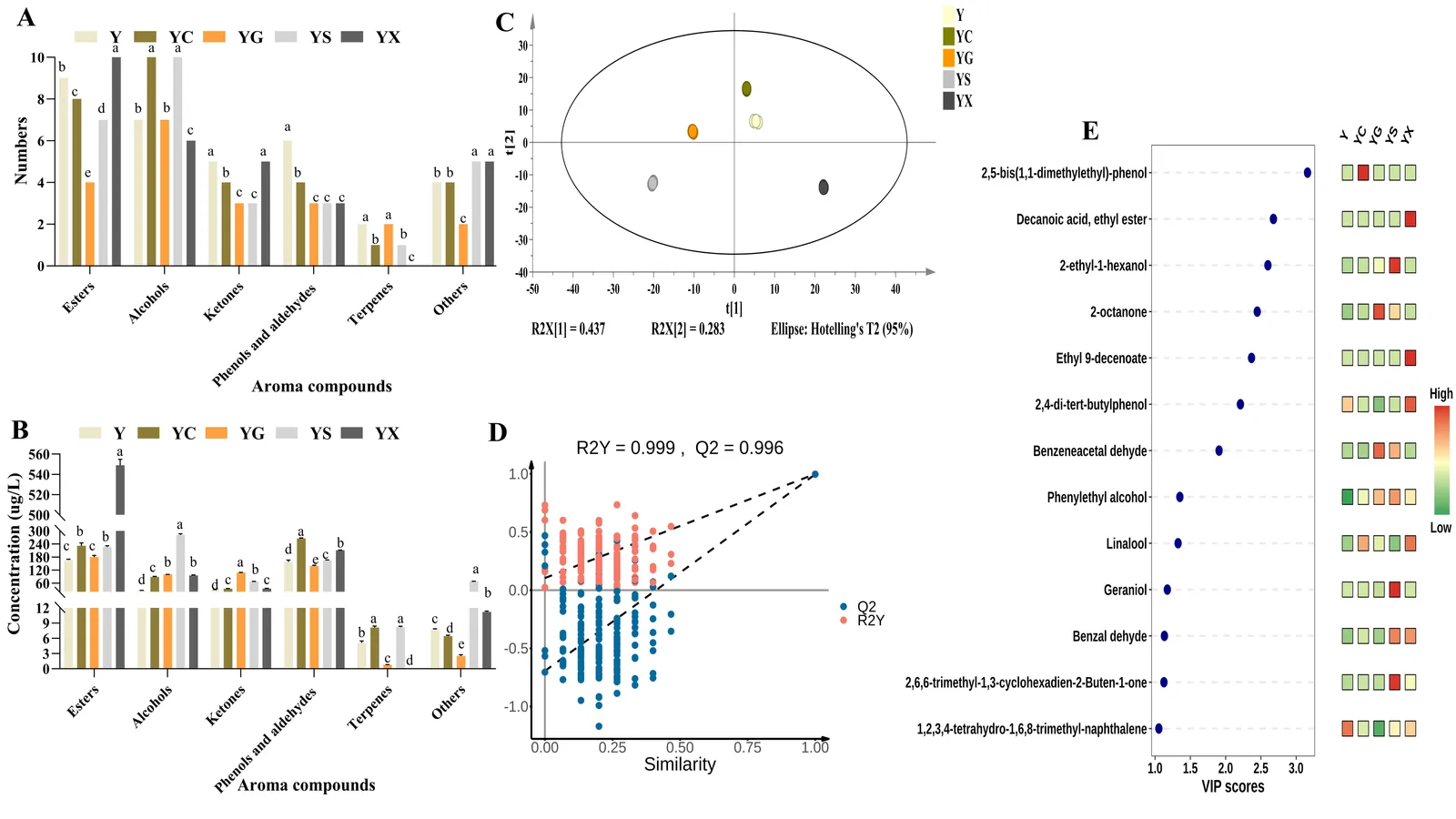
VOCs analysis by HS-SPME-GC-MS. (**A**) Comparison of the numbers of different VOCs. (**B**) Comparison of the concentration (μg/L) of different VOCs. (**C**) OPLS-DA score plot of different VOCs. (**D**) Sample replacement test diagram. (**E**) Differential VOCs VIP values (>1) and normalized heatmaps. Y: sterilized, unfermented control; YC: *B. longum* BL11; YG: *L. casei* LC15; YS: *S. thermophilus* LB42; YX: *L. plantarum* XJ25. a–e indicate statistical significance determined by one-way ANOVA (*p* < 0.05).

The VOCs in CPBs were analyzed via HS-SPME-GC-MS. The detailed information of the HS-SPME-GC-MS results is demonstrated in Table 1, including 21 esters, 18 alcohols, 8 ketones, 7 phenols and aldehydes, 6 terpenes and 10 other compounds. As shown in Figure 4A, the number of VOCs differed across CPB samples fermented with different strains. A total of 70 VOCs were obtained, with 33 VOCs in Y, 31 VOCs in YC, 21 VOCs in YG, 29 VOCs in YS and 29 VOCs in YX. Meanwhile, the YG displayed the lowest variety of VOCs, indicating that the flavor characteristics of the YG were poorer than those of the others. Among the various VOCs, esters were found to be predominant in CPB samples. The YX contained the highest number of esters (10 compounds). Alcohols were more abundant in the YC and YS (10 compounds each), whereas the YG had a higher number of terpenes (2 compounds). These results indicated that CPB fermented with different strains leads to the production of CPBs with different flavor properties. In terms of total VOCs content (Figure 4B), the YX had the highest levels (901.92 μg/L), followed by the YS (817.34 μg/L), the YC (633.32 μg/L), the YG (529.51 μg/L) and the Y (383.54 μg/L). Among the various VOCs detected, the YX contained the highest content of esters (548.92 μg/L). An OPLS-DA model was established based on the VOC concentrations in CPB samples fermented with different strains. This model displayed fantastic fit parameters (R2Y = 0.998, Q2 = 0.989) (Figure 4C). All groups of CPB samples were obviously independent and could be effectively discriminated from each other. CPB samples clustered obviously according to the strains, with distances of spatial separation reflecting compositional differences. Notably, the YX CPB sample displayed the greatest separation compared to the Y CPB sample, which reflected its distinctive aromatic properties. All CPB samples were located within the confidence intervals, and to ensure the OPLS-DA model was not overfit, a replacement test was employed (Figure 4D), which showed model validity without unfitting or overfitting. A variable importance in projection (VIP) value > 1 was used as the screening criterion for significant differences; 13 VOCs were identified as critical contributors (Figure 4E), such as decanoic acid ethyl ester, ethyl 9-decenoate, 2-ethyl-1-hexanol, benzeneacetaldehyde, 2-octanone, phenylethyl alcohol, linalool, geraniol, 2,4-di-tert-butylphenol, benzaldehyde, 2,5-bis (1,1-dimethylethyl)-phenol, and so on.

#### 3.3.3. GC-IMS Analysis

**Figure 5:**
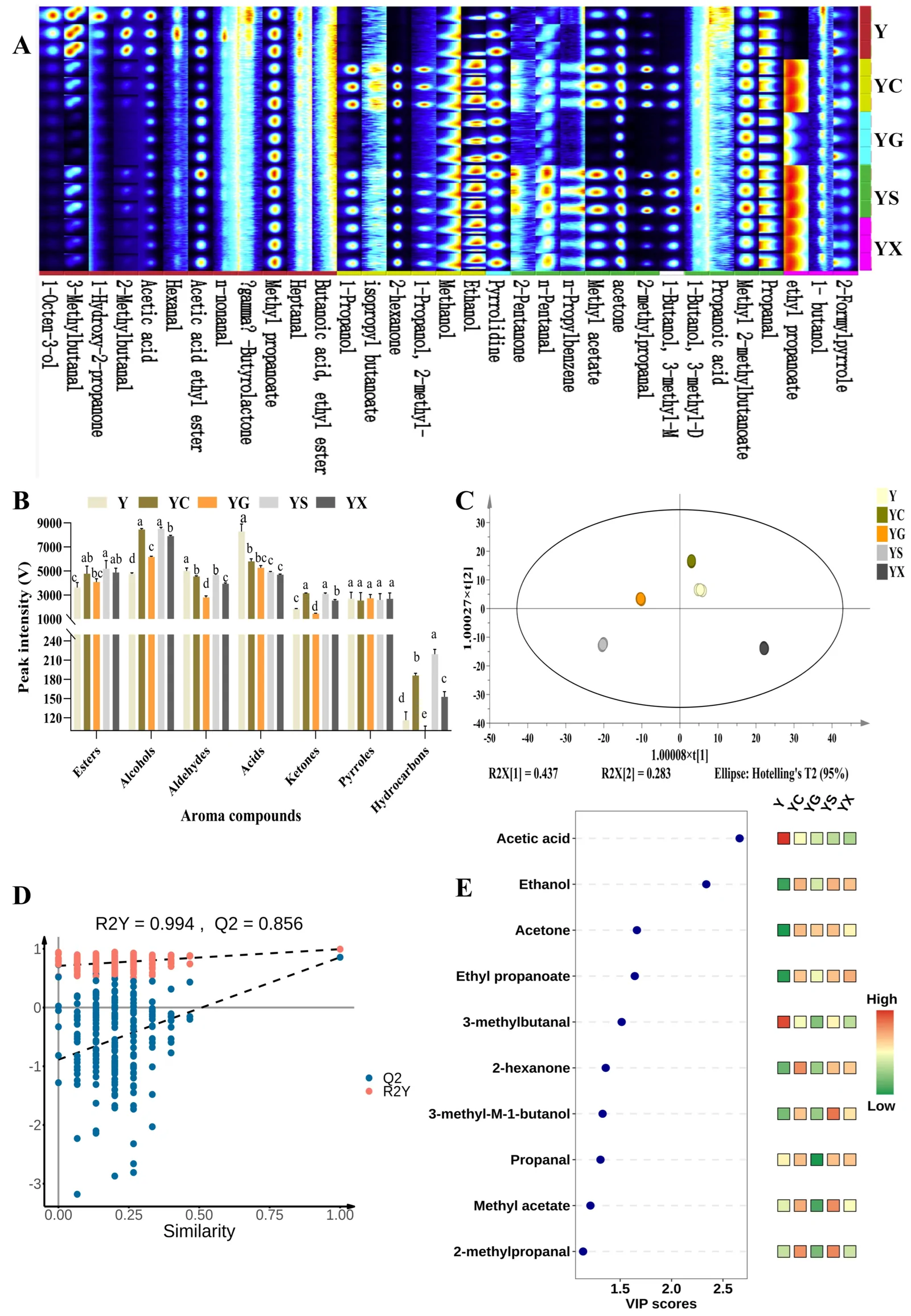
VOCs analysis by GC-IMS. (**A**) Fingerprints of different VOCs. (**B**) Comparison of the peak intensity of different VOCs. (**C**) OPLS-DA score plot of different VOCs. (**D**) Sample replacement test diagram. (**E**) Differential VOCs VIP values (>1) and normalized heatmaps. Y: sterilized, unfermented control; YC: *B. longum* BL11; YG: *L. casei* LC15; YS: *S. thermophilus* LB42; YX: *L. plantarum* XJ25. a–e indicate statistical significance determined by one-way ANOVA (*p* < 0.05).

In addition to HS-SPME-GC-MS, GC-IMS was also used to briefly identify VOCs and explore the impact of strains on the volatile characteristics of CPB. Most signals were observed within drift times of 1.0–1.5 ms and retention times of 200–400 s. To visualize strains-dependent differences in the VOCs of CPB samples, a fingerprint spectrum was established (Figure 5A). A total of 33 VOCs were identified in five CPB samples, including 8 esters, 8 alcohols, 8 aldehydes, 4 ketones, 2 acids, 2 pyrroles and 1 hydrocarbon. Among them, high peak levels were detected for 3-methylbutanal, 2-methylbutanal, methanol, propanal, and ethyl propanoate in the Y group. Notably, after fermentation, the highest signal intensity for volatiles was observed, including 1-propanol, acetic acid ethyl ester, 2-hexanone, 2-methyl-1-propanol, ethanol, 2-pentanone, n-pentanal, n-propylbenzene, methyl acetate, 2-methylpropanal, 3-methyl-1-butanol-D, 3-methyl-1-butanol-M, methyl 2-methylbutanoate, and ethyl propanoate. These results indicated that CPB samples fermented with different strains had their own characteristic VOC regions.

In terms of total VOC peak intensity (Figure 5B), the YC had the highest levels (29,408.33 ± 831.84), followed by the YS (29,134.06 ± 1149.55), the YX (26,733.91 ± 760.00), the Y (26,631.29 ± 434.38) and the YG (22,564.82 ± 526.92). Among the various VOCs detected, the YC (8441.01 ± 71.05) and YS (8501.75 ± 109.79) group contained the highest peak intensity of alcohols. An OPLS-DA model was established based on the VOCs’ peak intensities in CPB samples fermented with different strains. This model displayed fantastic fit parameters (R2Y = 0.994, Q2 = 0.856) (Figure 5C). All groups of CPB samples were obviously independent and could be effectively discriminated from each other. CPB samples clustered obviously according to the strains, with distances of spatial separation reflecting compositional differences. Notably, the YX and YG samples displayed the greatest separation, which reflected their distinctive aromatic properties compared to the Y sample. All CPB samples were located within the confidence intervals, and to ensure the OPLS-DA model was not overfit, a replacement test was employed (Figure 5D), which showed model validity without unfitting or overfitting. A VIP value > 1 was used as the screening criterion for significant differences, and 10 VOCs were identified as critical contributors (Figure 5E), including acetic acid, ethanol, acetone, ethyl propanoate, 3-methyl-butanal, 2-hexanone, 3-methyl-M-1-butanol, propanal, methyl acetate and 2-methylpropanal.

Flavor is one of the critical indicators for juice quality evaluation and consumer preference, formed by the interplay of various VOCs [45]. Currently, an increasing number of instrumental analysis techniques are used for the quantification and characterization of VOCs. For the analysis of VOCs, HS-SPME-GC-MS and GC-IMS technologies are mainly used. HS-SPME-GC-MS, a technique refined over years of development, now stands as a relatively mature analytical tool. Its strengths lie in the unparalleled variety of columns available, rapid separation efficiency, and robust qualitative and quantitative performance. GC-IMS, as a new food flavor analysis technology, has the advantages of sensitivity, rapidity and simple preprocessing in food [46]. A single analytical approach may miss some key VOCs, but the simultaneous use of multiple techniques can enhance the rigor of experimental results. Notably, the combination of HS-SPME-GC-MS and GC-IMS technologies has enabled the exhaustive characterization of VOCs. Table S2 displays the detection results of VOCs identified by GC-IMS in CPB samples. It is different from HS-SPME-GC-MS technology; a total of 33 VOCs were identified. In contrast, HS-SPME-GC-MS can detect more esters, alcohols and ketones, while GC-IMS enables the accurate detection and characterization of certain volatile monomers and dimers. From the HS-SPME-GC-MS analysis of VOCs, it was found that the esters, alcohols and ketone concentrations in CPB samples fermented with strains were the highest (Figure 4C). The result was similar to the findings reported by Xie et al. [47], who discovered that these concentrations were higher in fermented *Xuxiang* kiwifruit juice than in unfermented *Xuxiang* kiwifruit juice, enhancing the flavor of *Xuxiang* kiwifruit juice. Subsequently, the variations of esters, alcohols and ketones in CPB samples fermented with different strains were further studied.

Esters are of great industrial significance due to their absolutely low odor thresholds and dual ability to influence juice flavor—both directly and through complex synergistic interactions [48]. Ester compounds are one of the major VOCs in CPB samples, and they usually exhibit floral and fruity aromas that provide a pleasant odor to CPB. A total of 29 VOCs were detected in all CPB samples by HS-SPME-GC-MS and GC-IMS. The poorest variety of esters was observed in YG; there were only 12 kinds of esters, but there were 18 kinds of esters in YX (Table 1 and Table S2). Notably, 12 esters were newly generated after fermentation. Total ester concentration increased significantly from 167.40 ± 2.55 μg/L in Y to 548.92 ± 4.79 μg/L in YX, 180.22 ± 6.17 μg/L in YG, 226.65 ± 4.32 μg/L in YS and 232.07 ± 10.51 μg/L in YC. This trend is similar to the results detected by GC-IMS (Figure 5B). Additionally, methyl acetate, ethyl propanoate, ethyl 9-decenoate and decanoic acid ethyl ester were the key aroma-active compounds in CPB. The concentration of ethyl 9-decenoate, which contributes to the fat, milk and cheese odor [48], was much higher in YX samples.

Alcohols contribute significantly to the alcoholic sweetness of fermented juice and act as precursors for ester formation [49]. In general, the types and contents of alcohols in CPB samples showed an increasing trend after fermentation, with 12 new alcohols obtained. The prevailing alcohols found in CPB samples were 1-propanol, ethanol, 1-octen-3-ol, linalool, terpineol, 1-butanol, 2-ethyl-1-hexanol, phenylethyl alcohol and geraniol. Linalool is known for imparting sweet, rosy, floral and fruity odors, while phenylethyl alcohol displays floral, rose-like, sweet characteristics [50,51]. The highest content of linalool was found in YX samples with *L. plantarum* XJ25 fermentation. The result is consistent with the findings reported by Yao et al. [52], who discovered that *L. plantarum* can produce linalool. The content of geraniol was notably increased after fermentation (*p* < 0.05) except for the YG samples, imparting an intense rose and citrus characteristic to the fermented CPB samples [35]. Phenylethyl alcohol was produced via either glycoside hydrolysis or the shikimate biosynthesis pathway and is associated with the odors of fresh bread, roses, and sweet flowers [46]. The content of phenylethyl alcohol increased significantly after fermentation. It is worth noting that 1-octen-3-ol exhibits a mushroom odor, representing the highest peak intensity in unfermented CPB at a concentration of 617.18 ± 175.24. In summary, the odorant profile of CPB’s alcohols was enhanced with fermentation, imparting a milder fruity and floral character.

Ketones are generated via amino acid degradation and lipid oxidation [53] and play a pivotal role in the flavor characteristics of juice products. A total of 12 VOCs were obtained in the different CPB samples by HS-SPME-GC-MS and GC-IMS; six ketones were detected in the Y sample by HS-SPME-GC-MS, with four additional ketones identified via GC-IMS. Post-fermentation, the number of GC-MS-detected ketones decreased to 1–4 depending on the strain. Total ketones concentration increased significantly from 29.02 ± 1.12 μg/L in Y to 34.48 ± 0.83 μg/L in YC, 108.64 ± 0.85 μg/L in YG and 67.55 ± 0.47 μg/L in YS, whereas YX showed no significant change. This is similar to results reported by Guan et al. [28] who reported the same trend of significant increases in ketones with fermentation in fruit juice. In addition, acetone, 2-octanone and 2-hexanone were the key aroma-active compounds in CPB. Of these, 2-hexanone, which contributes a cheesy, banana and cinnamon odor [51], was detected at significantly higher relative abundance in CPB samples with fermentation. Due to their low perception threshold, they played a vital role in CPB samples.

### 3.4. Non-Volatile Metabolites Analysis

**Figure 6:**
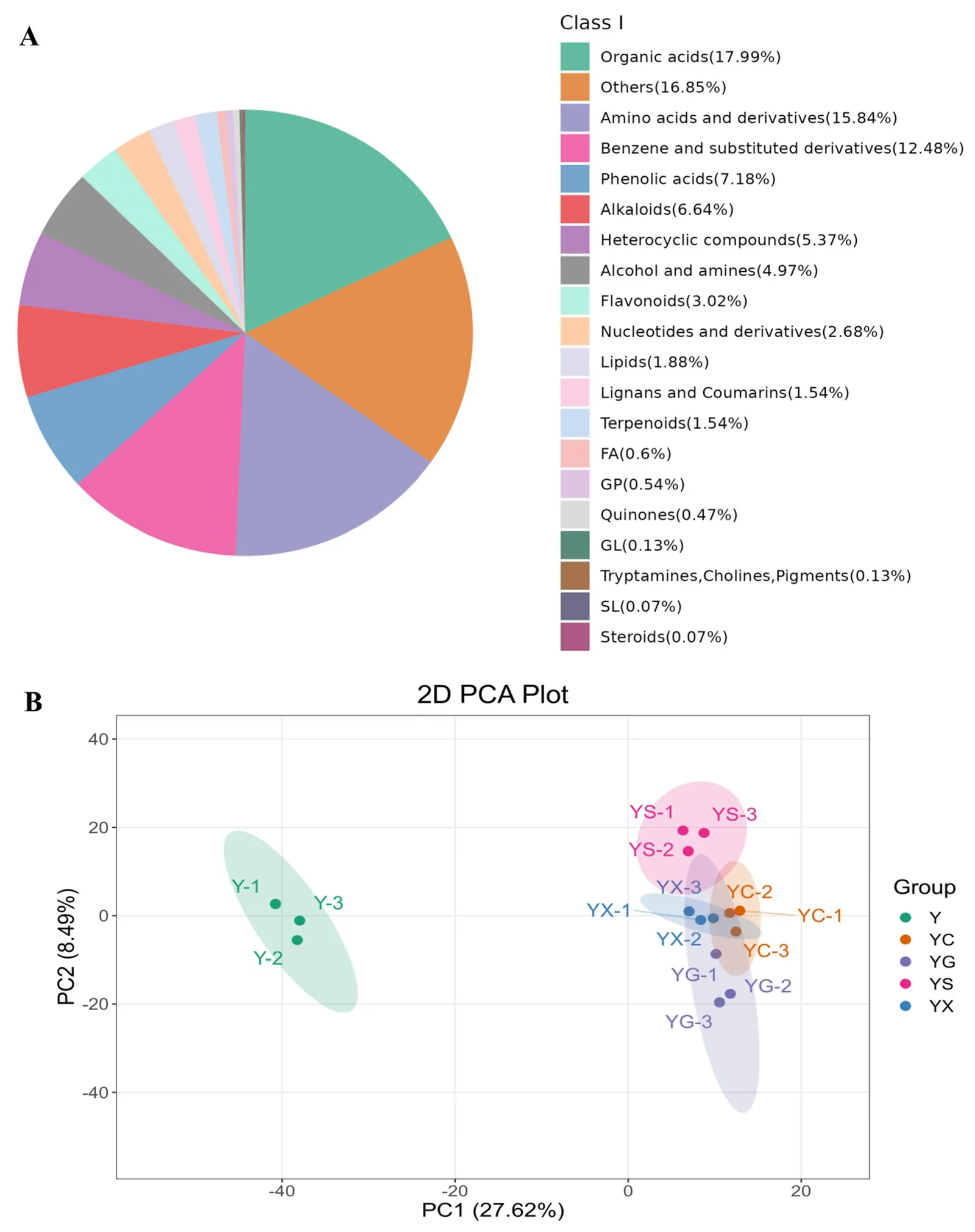
Non-volatile metabolites analysis by UPLC-MS/MS. (**A**) Classification of non-volatile metabolites. (**B**) PCA. Y: sterilized, unfermented control; YC: *B. longum* BL11; YG: *L. casei* LC15; YS: *S. thermophilus* LB42; YX: *L. plantarum* XJ25. Note: Round individual values to two decimal places to obtain the sum of percentages.

In this study, a total of 1490 non-volatile metabolites were obtained, with 936 in positive ion mode and 554 in negative ion mode. These metabolites were categorized into several classes (Figure 6A), including organic acids (17.99%), others (16.85%), amino acids and derivatives (15.84%), benzene and substituted derivatives (12.48%), phenolic acids (7.18%), alkaloids (6.64%), heterocyclic compounds (5.37%), alcohol and amines (4.97%), flavonoids (3.02%), nucleotides and derivatives (2.68%), lipids (1.88%), lignans and coumarins (1.54%) and terpenoids (1.54%). The variations in non-volatile metabolites were affected by the differences in strains and their unique metabolic pathways. The experiments were performed using PCA chemometrics to obtain further insight into the metabolic differences between unfermented samples and fermented CPB samples (Figure 6B). The first and second principal components, PC1 and PC2, accounted for 8.49% and 27.62% of the overall variance, respectively. The samples between the groups were clustered, and unfermented CPB was clearly distinguishable from fermented CPB, suggesting that there are obvious differences among the five CPB samples in terms of their metabolic profiles. The volcano plots display the differences in the relative content of non-volatile metabolites between each CPB sample. There were 155 (101 up, 54 down), 154 (99 up, 55 down), 121 (83 up, 38 down) and 123 (79 up, 44 down) differential metabolites between YC vs. Y, YG vs. Y, YS vs. Y and YX vs. Y, respectively (Figure S1). Based on analysis of the KEGG database, metabolic pathway enrichment analysis was conducted on the differential metabolites before and after fermentation (Figure S2). The top 20 metabolic pathways were obtained and the differential metabolites were functionally enriched in different pathways. The YC, YG, YS and YX exhibited high similarity in categories of their metabolic pathway, primarily involving the phosphotransferase system, galactose metabolism, ascorbate and aldarate metabolism, phenylalanine metabolism, phosphonate and phosphinate metabolism, vancomycin resistance, and cysteine and methionine metabolism. However, the enrichment ratios of specific pathways varied significantly among the different strains’ fermentation. For example, the YC vs. Y showed significant enrichment in the methane metabolism pathway, while the YG vs. Y exhibited differences primarily in the phosphotransferase system. Based on the KEGG enrichment pathway analysis, galactose metabolism was the most important metabolic pathway. Galactose metabolism is related to lactose production, specifically to the production of β-Gal, GalK, PK and GK and other related enzymes [16,54,55,56].

**Figure 7:**
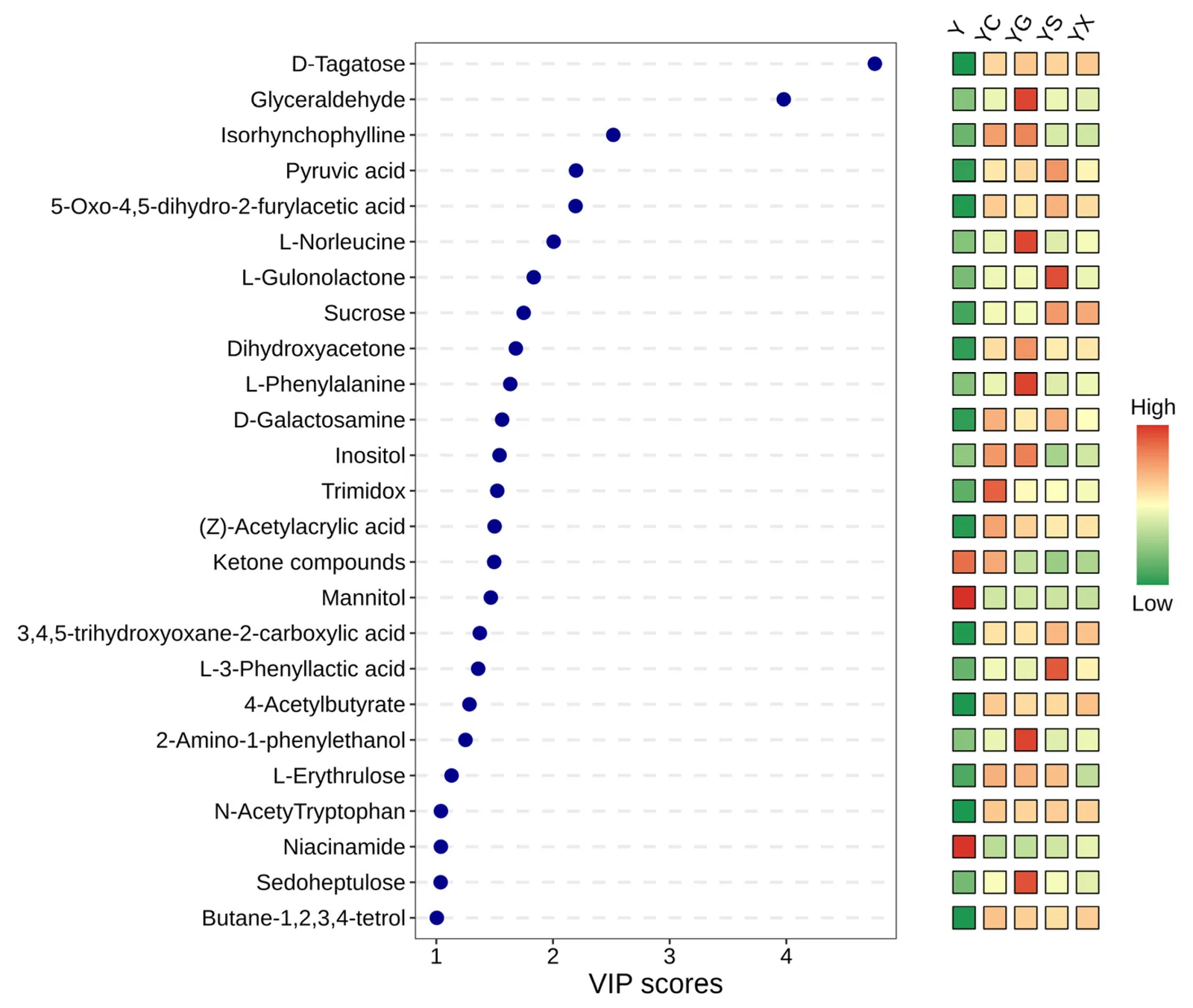
Differential non-volatile metabolites VIP values (>1) and normalized heatmaps.

Relationships between differential metabolites in each group are displayed in a Venn diagram (Figure S3); a total of 113 non-volatile metabolites were obtained. A VIP value > 1 was used as the screening criterion for significant differences; 25 key metabolites were identified as vital contributors, which were used for cluster analysis subsequently (Figure 7). Heatmap analysis exhibited a relative increase in the abundance of 22 metabolites, whereas 3 metabolites revealed a relative decrease in abundance. These metabolites included 9 saccharides, 5 organic acids, 3 amino acids and derivatives, 2 ketone compounds, 2 alkaloids, 1 alcohol and amines, 1 phenolic acid, 1 lactone, and 1 vitamin.

The content and composition of organic acids play vital roles in the preservation and odor characteristics of fruit products [57]. Pyruvic acid is a precursor to numerous chemical compounds, including hormones, pigments, terpenoids, and amino acids [58]. Based on the results of our study (Figure S2), the highest levels of pyruvic acid were detected in YS, followed by YG, YC and YX. L-lactic acid was the predominant organic acid produced after fermentation. The highest levels of L-lactic acid were found in YG with *L. casei* fermentation. This is similar to results reported by Muhialdin et al. [59], who reported that lactic acid is much higher in pineapple juice with *L. casei* ATCC334 fermentation. The strains possess aldolase, an enzyme that catalyzes the conversion of glucose to lactic acid [60]. This is similar to the results of the above experiments, which demonstrated a decrease in glucose content after fermentation. Lactic acid is predominantly employed in the juice sector, where its application not only enhances the overall flavor profile but also inhibits the growth of pathogenic microorganisms [61]. Additionally, lactic acid facilitates the accumulation of pigmented substances, thereby contributing to the enhanced redness of fermented CPB [62]. As for amino acids and derivatives, the results showed that L-phenylalanine was an important functional metabolite. Phenylalanine, as a pivotal precursor of ester flavor compounds, constitutes a critical pathway for flavor synthesis [63]. The content of L-phenylalanine increased in CPB fermented with different strains, especially with a significant increase observed in the YG group (*p* < 0.05).

### 3.5. Correlation Network Heatmap Between Non-Volatile Compounds and Flavor Compounds

**Figure 8:**
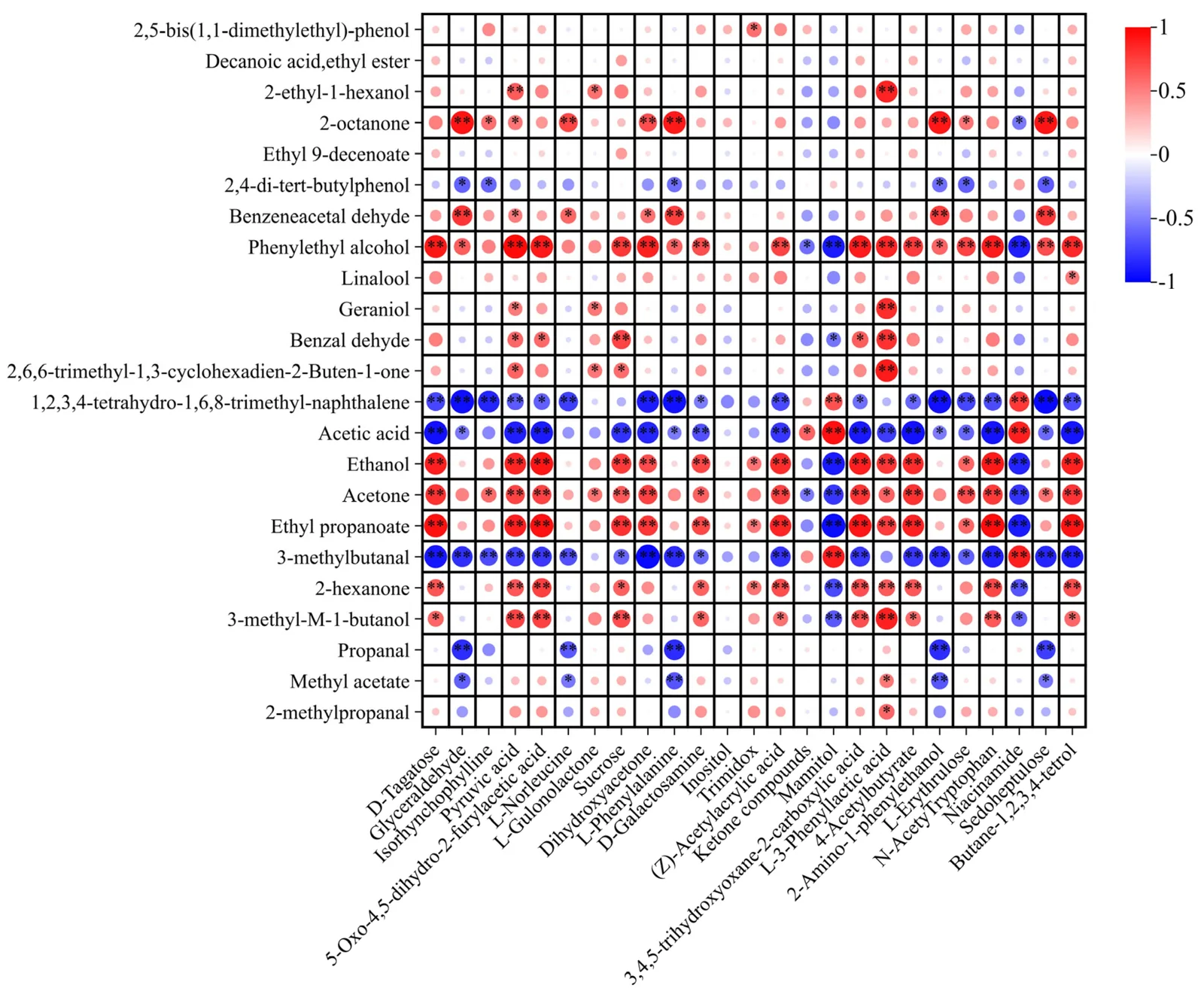
Correlation analysis between non-volatile metabolites and VOCs in CPB samples fermented by different strains, VOCs obtained by HS-SPME-GC-MS and GC-IMS. * *p* < 0.05, ** *p* < 0.01, indicate significant difference. Red and blue indicate positive and negative correlation, while color depth indicates the degree of correlation.

Given that non-volatile metabolites may be precursors of volatile flavor compounds, the correlation of 23 key differential VOCs and 25 shared differential non-volatile metabolites was analyzed (Figure 8). Most metabolites exhibited significant positive correlations with 2,5-bis (1,1-dimethylethyl)-phenol, 2-ethyl-1-hexanol, benzeneacetaldehyde, geraniol, 2,6,6-trimethyl-1,3-cyclohexadien-2-buten-1-one and 2-methylpropanal, whereas they were significantly negatively correlated with 2,4-di-tert-butylphenol and propanal (*p* < 0.05). In addition, phenylethyl alcohol established 20 significant correlations with 25 metabolites, including 3 negative correlations and 17 positive correlations. Conversely, 3-methylbutanal also established 20 significant correlations, including 2 negative correlations and 18 positive correlations. This result indicated possible crosstalk between VOCs and non-volatile metabolites in CPBs, which is regulated by enriched metabolic pathways.

### 3.6. Sensory Evaluation

**Figure 9:**
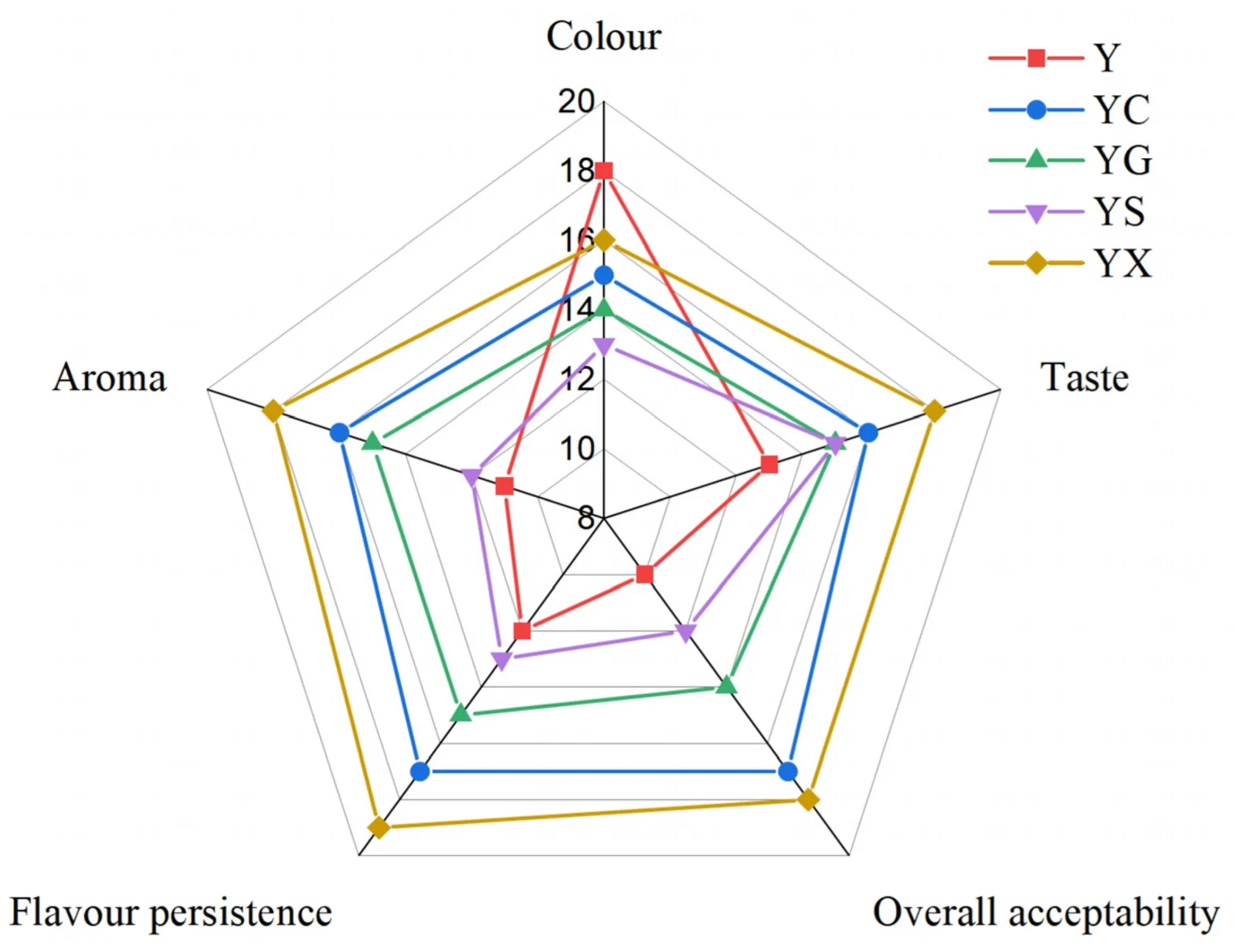
Sensory evaluation of the fermented CPB.

The sensory evaluation results of CPB fermentation by different strains are displayed in Figure 9. Sensory evaluation methods offer a direct measure of the acceptance of fermented CPB’s quality and taste by the targeted population [64]. Compared with the unfermented Y sample, the taste, odor, flavor and overall acceptability of CPB samples were improved with fermentation, respectively, while the color properties of CPB show a downward trend. Among them, the YX sample had the highest overall score for its pleasant taste, flavor persistence, flavor and overall acceptability. The juice flavor score is readily observable, attributing to the enhanced production of VOCs by strains during fermentation, consistent with prior studies [28], indicating that CPB sample fermented with *L*. *plantarum* XJ25 had the potential to improve in aroma and taste, which can be mechanistically attributed to its unique VOC profile. The decanoic acid ethyl ester, ethyl 9-decenoate, linalool, ethanol and ethyl propanoate showed the strongest positive correlations with taste, aroma, overall acceptability and flavor persistence (Figure S4). The higher ester content in *L. plantarum*-fermented CPB likely explains their higher sensory acceptability scores.

## 4. Conclusions

CP is known for its rich nutrients and bioactive components. However, a large amount of CP is discarded without treatment, leading to the waste of resources and serious environmental problems. This study investigated the effects of different strains on the flavor characteristics, metabolic characteristics, and antioxidant activity of CPB. The findings revealed that all four strains exhibited excellent growth potential in CPB. Fermentation reduced the sugar content, increased the levels of antioxidant activity, and further changed the metabolic profile of CPB. Fermentation could also promote the production of VOCs, including esters, alcohols and ketones, thereby improving the flavor quality. CPB had the best aroma and overall acceptance after *L*. *plantarum* XJ25 fermentation. Therefore, *L. plantarum* XJ25 can be used as a potential starter culture for CPB. Future studies should investigate the effects of co-culture and mixed-strain fermentation, as well as the optimization of key fermentation parameters, to further enhance the metabolic potential of fermentation in CP.

## Figures and Tables

**Table 1:** Volatile flavor profiles in CPB fermented with strains by GC-MS (μg/L).

Aroma Compounds	CAS	Concentration (μg/L)				
		Y	YC	YG	YS	YX
**Esters**						
Ethyl 9-decenoate	067233-91-4	ND	ND	ND	ND	113.49 ± 1.17 ^a^
Decanoic acid, ethyl ester	000110-38-3	ND	ND	ND	ND	145.30 ± 1.35 ^a^
Dodecanoic acid, ethyl ester	000106-33-2	ND	ND	ND	ND	10.63 ± 0.70 ^a^
Ethyl 9-hexadecenoate	054546-22-4	ND	ND	ND	ND	16.63 ± 0.50 ^a^
Hexadecanoic acid, ethyl ester	000628-97-7	4.05 ± 0.41 ^a^	ND	ND	ND	ND
Octanoic acid, ethyl ester	000106-32-1	153.32 ± 2.90 ^e^	216.29 ± 10.21 ^b^	175.29 ± 5.97 ^d^	189.62 ± 6.82 ^c^	252.67 ± 2.87 ^a^
2,2,4-Trimethyl-1,3-pentanediol diisobutyrate	006846-50-0	3.82 ± 0.15 ^c^	6.34 ± 0.65 ^a^	2.45 ± 0.12 ^d^	5.45 ± 0.48 ^b^	0.48 ± 0.08 ^e^
Tetradecanoic acid, ethyl ester	000124-06-1	0.14 ± 0.01 ^a^	ND	ND	ND	ND
Phthalic acid, hex-3-yl isobutyl ester	1000356-95-4	0.42 ± 0.07 ^b^	ND	ND	3.21 ± 0.03 ^a^	ND
Methyl salicylate	000119-36-8	2.22 ± 0.37 ^c^	1.67 ± 0.22 ^d^	ND	8.88 ± 0.48 ^a^	4.01 ± 0.48 ^b^
Phthalic acid, butyl isohexyl ester	1000309-03-6	0.51 ± 0.01 ^a^	ND	ND	ND	ND
Formic acid, heptyl ester	000112-23-2	2.39 ± 0.09 ^a^	ND	1.25 ± 0.05 ^b^	ND	ND
1,2-Benzenedicarboxylic acid, bis(2-methylpropyl) ester	000084-69-5	0.53 ± 0.01	ND	ND	ND	ND
Propanoic acid, 2-methyl-, 3-hydroxy-2,2,4-trimethylpentyl ester	000077-68-9	ND	0.74 ± 0.04 ^c^	1.22 ± 0.08 ^b^	7.90 ± 0.85 ^a^	ND
Phthalic acid, 7-bromoheptyl isobutyl ester	1000415-51-7	ND	0.40 ± 0.05 ^a^	ND	ND	ND
Ethyl 2-(5-methyl-5-vinyltetrahydrofuran-2-yl)propan-2-yl carbonate	1000373-80-3	ND	3.68 ± 0.03 ^c^	ND	7.09 ± 0.02 ^a^	4.77 ± 0.33 ^b^
Phthalic acid, isobutyl trans-hex-3-enyl ester	1000360-48-1	ND	0.52 ± 0.03 ^a^	ND	ND	ND
Geranyl formate	000105-86-2	ND	2.42 ± 0.09 ^a^	ND	ND	ND
Di-sec-butyl phthalate	1000373-65-4	ND	ND	ND	ND	0.54 ± 0.03 ^a^
Phthalic acid, 8-bromooctyl isobutyl ester	1000382-55-3	ND	ND	ND	ND	0.40 ± 0.02 ^a^
Dibutyl phthalate	000084-74-2	ND	ND	ND	6.85 ± 0.15 ^a^	ND
**Alcohols**						
3-ethyl-4-methylpentan-1-ol	038514-13-5	1.15 ± 0.08 ^a^	ND	ND	ND	ND
2-ethyl-1-hexanol	000104-76-7	ND	8.41 ± 0.22 ^d^	33.68 ± 0.84 ^b^	147.26 ± 1.26 ^a^	10.02 ± 0.26 ^c^
1-heptanol	000111-70-6	ND	3.52 ± 0.06 ^b^	2.01 ± 0.07 ^c^	19.53 ± 0.26 ^a^	ND
1-phenoxypropan-2-ol	000770-35-4	ND	ND	ND	4.94 ± 0.16 ^a^	ND
2-((2S,4aR)-4a,8-dimethyl-1,2,3,4,4a,5,6,7-octahydronaphthalen-2-yl)propan-2-ol	117066-77-0	ND	ND	ND	1.35 ± 0.00 ^a^	ND
Linalool	000078-70-6	13.72 ± 1.03 ^d^	34.95 ± 1.54 ^b^	21.12 ± 0.32 ^c^	11.54 ± 0.22 ^e^	41.07 ± 1.64 ^a^
(R)-3,7-dimethyl-6-octen-1-ol	001117-61-9	2.45 ± 0.13 ^a^	ND	ND	ND	ND
(E)-3,7,11-trimethyl-1,6,10-dodecatrien-3-ol	040716-66-3	ND	2.48 ± 0.04 ^c^	ND	17.73 ± 0.10 ^a^	3.29 ± 0.01 ^b^
3,7,11-trimethyl-6,10-dodecadien-1-ol	051411-24-6	1.53 ± 0.01 ^a^	ND	ND	ND	ND
Terpineol	1000411-59-6	3.50 ± 0.03 ^c^	9.56 ± 0.45 ^b^	ND	ND	15.44 ± 0.24 ^a^
Phenylethyl alcohol	000060-12-8	1.92 ± 0.06 ^e^	20.00 ± 1.00 ^d^	29.18 ± 0.57 ^b^	33.33 ± 1.89 ^a^	23.82 ± 0.64 ^c^
Geraniol	000106-24-1	1.58 ± 0.01 ^d^	1.84 ± 0.06 ^b^	ND	26.45 ± 0.49 ^a^	1.72 ± 0.01 ^c^
Nerolidol	000142-50-7	ND	1.96 ± 0.01 ^a^	ND	ND	ND
(Z)-3,7-dimethyl-2,6-octadien-1-ol	000106-25-2	ND	0.81 ± 0.02 ^a^	ND	ND	ND
L-α-terpineol	010482-56-1	ND	ND	7.61 ± 0.12 ^b^	9.97 ± 0.40 ^a^	ND
trans-Linalool oxide (furanoid)	034995-77-2	ND	ND	2.48 ± 0.10 ^a^	ND	ND
(Z)-3,7-dimethyl-2,6-octadien-1-ol	000106-25-2	ND	ND	ND	16.63 ± 0.34 ^a^	ND
Cis-5-trimethyl-α,α,5-ethenyltetrahydro-2-Furanmethanol	005989-33-3	ND	5.24 ± 0.15 ^a^	3.96 ± 0.19 ^b^	ND	ND
**Ketones**						
2,6,6-trimethyl-1,3-cyclohexadien-2-Buten-1-one	023726-93-4	23.25 ± 0.97 ^a^	10.95 ± 0.33 ^d^	2.65 ± 0.02 ^e^	14.24 ± 0.04 ^c^	16.67 ± 0.14 ^b^
1-(1H-pyrrol-2-yl)-ethanone	001072-83-9	4.23 ± 0.13 ^a^	ND	2.72 ± 0.01 ^b^	ND	2.89 ± 0.01 ^b^
1-(2-pyridinyl)-1-propanone	003238-55-9	0.33 ± 0.05 ^a^	ND	ND	ND	ND
4-(2,6,6-trimethyl-1-cyclohexen-1-yl)-3-buten-2-one	014901-07-6	0.48 ± 0.01 ^a^	ND	ND	ND	ND
2-octanone	000111-13-7	ND	12.19 ± 0.22 ^d^	103.26 ± 0.85 ^a^	50.87 ± 0.44 ^b^	14.43 ± 0.43 ^c^
1-(2,6,6-trimethyl-1,3-cyclohexadien-1-yl)-2-buten-1-one	023696-85-7	0.73 ± 0.01 ^a^	10.92 ± 0.31 ^a^	ND	ND	0.47 ± 0.04 ^b^
2,6-bis(1,1-dimethylethyl)-2,5-cyclohexadiene-1,4-dione	000719-22-2	ND	0.41 ± 0.03 ^b^	ND	ND	0.57 ± 0.00 ^a^
Megastigmatrienone	038818-55-2	ND	ND	ND	2.45 ± 0.01 ^a^	ND
**Phenols and aldehydes**						
Benzeneacetaldehyde	000122-78-1	4.23 ± 0.09 ^c^	ND	64.46 ± 0.98 ^a^	44.77 ± 0.34 ^b^	4.69 ± 0.40 ^c^
Nonanal	000124-19-6	6.24 ± 0.14 ^a^	ND	ND	ND	ND
5-methyl-2-furancarboxal dehyde	000620-02-0	3.59 ± 0.14 ^a^	ND	ND	ND	ND
Benzal dehyde	000100-52-7	2.40 ± 0.08 ^d^	7.35 ± 0.40 ^b^	3.31 ± 0.28 ^c^	22.67 ± 1.25 ^a^	21.06 ± 0.25 ^a^
Decanal	000112-31-2	1.64 ± 0.09 ^a^	0.75 ± 0.05 ^b^	ND	ND	ND
2,4-di-tert-butylphenol	000096-76-4	139.53 ± 6.49 ^b^	100.26 ± 0.70 ^c^	71.45 ± 2.91 ^d^	97.67 ± 2.05 ^c^	185.65 ± 0.32 ^a^
2,5-bis(1,1-dimethylethyl)-phenol	005875-45-6	ND	157.57 ± 2.12 ^a^	ND	ND	ND
**Terpenes**						
β-ocimene	013877-91-3	ND	ND	ND	6.88 ± 0.13 ^a^	ND
2,6-dimethyl-2,4,6-octatriene	000673-84-7	0.71 ± 0.04 ^a^	ND	ND	ND	ND
1,7,7-trimethyl-bicyclo[2.2.1]hept-2-ene	000464-17-5	ND	8.13 ± 0.24 ^a^	ND	ND	ND
Styrene	000100-42-5	ND	ND	0.03 ± 0.00 ^a^	ND	ND
1,3,5,7-cyclooctatetraene	000629-20-9	ND	ND	0.74 ± 0.04 ^b^	1.34 ± 0.01 ^a^	ND
3-carene	013466-78-9	4.39 ± 0.30 ^a^	ND	ND	ND	ND
**Others**						
2,5-di-tert-butyl-1,4-benzoquinone	002460-77-7	0.67 ± 0.08 ^c^	1.18 ± 0.10 ^b^	ND	ND	1.68 ± 0.03 ^a^
1,2,3,4-tetrahydro-1,1,6-trimethyl-naphthalene	000475-03-6	2.13 ± 0.03 ^b^	ND	ND	ND	2.55 ± 0.03 ^a^
1,2,3,4-tetrahydro-1,6,8-trimethyl-naphthalene	030316-36-0	ND	1.55 ± 0.03 ^c^	0.62 ± 0.04 ^d^	21.56 ± 0.07 ^a^	5.04 ± 0.23 ^b^
2,3-dihydro-1,1,5,6-tetramethyl-1H-iNDene	000942-43-8	ND	ND	ND	ND	0.68 ± 0.05 ^a^
2-(2-butenyl)-1,3,5-trimethyl-benzene	063435-25-6	ND	ND	ND	6.66 ± 0.31 ^a^	ND
3-methoxy-benzenamine	000536-90-3	3.98 ± 0.02 ^a^	ND	ND	ND	ND
O-aminobenzohydroxamic acid	005623-04-1	ND	1.66 ± 0.02 ^a^	ND	ND	ND
tert-butyl-p-benzoquinone	003602-55-9	ND	ND	ND	16.20 ± 0.02 ^a^	ND
Benzothiazole	000095-16-9	ND	ND	ND	5.22 ± 0.16 ^a^	ND
Decamethyl-cyclopentasiloxane	000541-02-6	0.98 ± 0.10 ^d^	2.05 ± 0.12 ^b^	1.90 ± 0.17 ^b^	19.03 ± 0.08 ^a^	1.27 ± 0.02 ^c^
Total		383.54 ± 9.21 ^e^	633.32 ± 11.37 ^c^	529.51 ± 4.35 ^d^	817.34 ± 5.99 ^b^	901.92 ± 8.54 ^a^

Note: Y: sterilized, unfermented control; YC: *B. longum* BL11; YG: *L. casei* LC15; YS: *S. thermophilus* LB42; YX: *L. plantarum* XJ25. ^a^^–e^ indicate statistical significance determined by one-way ANOVA (*p* < 0.05). ND: not detected.

## Data Availability

The original contributions presented in this study are included in the article/Supplementary Materials. Further inquiries can be directed to the corresponding authors.

## Supplementary Materials

The following supporting information can be downloaded at: https://www.mdpi.com/article/10.3390/microorganisms14081839/s1, Table S1: Main response substances corresponding to sensors; Table S2: Volatile flavor profiles in CPB fermented with LAB by GC-IMS (%); Figure S1: Volcano plot of differential functional metabolites among compared groups (YC vs. Y, YG vs. Y, YS vs. Y, YX vs. Y) obtained by UPLC-MS/MS; Figure S2: Bubble plots of the top 20 KEGG pathways for differential functional metabolites among compared groups (YC vs. Y, YG vs. Y, YS vs. Y, YX vs. Y) obtained by UPLC-MS/MS; Figure S3: Venn plots of non-volatile metabolites in 5 CPB samples obtained by UPLC-MS/MS, the numbers represent the number of non-volatile compounds in each comparison group, and the overlap represent the number of non-volatile compounds common to multiple groups; Figure S4: Correlation analysis between sensory evaluation and VOCs in CPB samples fermented by different strains.
