# Supplementary material for: Fermentation Improves the Antioxidant Activity, Flavor Components and Metabolic Characteristics of Coffee Pulp Beverage
Source: Microorganisms. 2026 Aug 19;14(8):1839. doi: 10.3390/microorganisms14081839 (PMC13515781; doi:10.3390/microorganisms14081839)
Supplement: Supplementary file 1 [file microorganisms-14-01839-s001.zip › microorganisms-4495989-supplementary.pdf]

Table S1 Main response substances corresponding to sensors

Sensors	Substances for sensing	Concentration Range
Sn_1	Alkanes, Smoke	200 ~ 10000 ppm
Sn_2	Alcohols, Aldehydes, Short chain alkanes	1 ~ 1000 ppm
Sn_3	Ozone	10 ~ 1000 ppm
Sn_4	Sulfide, Hydrogen sulfide	1 ~ 200 ppm
Sn_5	Nitride, Ammonia	5 ~ 500 ppm
Sn_6	Organic gases, Benzoncs, Aldehydes, Aromatic compounds	5 ~ 500 ppm
Sn_7	Short-chain alkanes, Natural gas, Biogas	300 ~ 10000 ppm
Sn_8	Short chain alkanes	300 ~ 10000 ppm
Sn_9	Short chain alkanes	0 ~ 500 ppm
Sn_10	Hydrogen	100 ~ 1000 ppm
Sn_11	Allyl sulfide	300 ~ 10000 ppm
Sn_12	Ketones, Alcohols	300 ~ 10000 ppm
Sn_13	Methane	300 ~ 10000 ppm
Sn_14	Combustible gases	300 ~ 10000 ppm

Table S2 Volatile flavor profiles in CPB fermented with LAB by GC-IMS (%).

NO	Aroma compounds	CAS	Concentration (%)				
			Y	YC	YG	YS	YX
	**Esters**						
1	Methyl propanoate	C554121	1462.39±118.86	1250.94±248.73	1366.45±99.57	1393.95±230.62	1359.34±139.94
2	Acetic acid ethyl ester	C141786	911.59±190.75	664.12±303.51	709.96±112.75	820.05±330.66	728.15±174.95
3	ethyl propanoate	C105373	87.50±20.60	1436.52±26.23	1054.25±7.20	1469.96±3.64	1584.83±5.26
4	Methyl acetate	C79209	314.13±22.30	527.93±0.85	49.58±4.18	589.37±25.00	358.96±10.20
5	isopropyl butanoate	C638119	17.94±2.45	26.02±0.69	19.88±0.90	21.46±1.23	22.40±3.05
6	Methyl 2-methylbutanoate	C868575	200.29±15.94	286.77±7.36	301.87±10.65	310.25±29.79	249.15±9.85
7	Butanoic acid, ethyl ester	C105544	109.89±3.14	101.46±3.04	107.80±4.93	99.66±4.97	97.59±4.33
8	gamma -Butyrolactone	C96480	516.48±32.91	476.45±20.97	463.10±24.23	476.19±25.65	468.24±15.28
	**Alcohols**						
9	1-Propanol, 2-methyl-	C78831	84.94±5.54	138.04±5.19	84.29±3.18	112.61±4.76	124.02±3.77
10	1-Propanol	C71238	133.82±4.39	455.76±3.43	134.29±8.02	358.26±11.93	336.66±6.88
11	1-Butanol, 3-methyl-M	C123513	114.53±3.04	715.12±9.25	207.20±19.48	979.37±7.65	617.62±20.00
12	1-Butanol, 3-methyl-D	C123513	439.18±14.21	461.86±4.25	447.02±13.51	467.08±18.27	429.24±16.49
13	Ethanol	C64175	1325.34±87.92	4170.74±27.42	2871.95±6.88	4171.46±46.18	4013.15±7.83
14	1-Octen-3-ol	C3391864	617.18±175.24	217.85±15.74	170.59±12.63	138.27±9.68	145.50±21.48
15	Methanol	C67561	1909.71±101.08	2148.76±16.85	2133.78±17.31	2145.36±92.44	2095.89±27.20
16	1- butanol	C71363	127.36±4.75	132.88±7.32	121.32±4.15	129.33±2.87	132.32±12.90
	**Aldehydes**						
17	2-methylpropanal	C78842	225.72±21.37	550.77±66.87	82.92±2.88	573.14±24.35	239.24±20.92
18	Propanal	C123386	1857.14±15.85	1985.43±20.62	1357.63±67.56	1981.32±49.45	1975.73±17.83
19	2-Methylbutanal	C96173	518.87±52.90	374.10±6.62	133.69±8.77	398.20±16.61	297.86±0.26

20	3-Methylbutanal	C590863	1118.92±33.56	479.67±13.47	106.59±0.43	545.61±15.64	293.46±10.80
21	n-Pentanal	C110623	48.12±4.78	49.37±0.70	44.50±3.50	70.94±3.04	59.34±5.96
22	n-nonanal	C124196	989.42±63.24	897.90±15.05	882.07±15.34	903.80±30.12	879.30±19.11
23	Heptanal	C111717	141.71±5.46	143.46±4.91	132.88±1.09	128.51±5.53	123.12±4.18
24	Hexanal	C66251	95.27±10.13	61.06±1.15	60.13±2.47	64.65±2.70	60.29±6.08
	**Ketones**						
25	2-hexanone	C591786	168.02±9.41	1081.06±5.77	328.32±1.96	890.07±15.47	856.35±19.53
26	1-Hydroxy-2-propanone	C116096	353.29±100.68	207.51±10.72	193.43±1.92	196.33±9.20	190.61±3.54
27	acetone	C67641	1308.95±83.06	1830.29±27.90	905.90±38.14	1974.56±71.19	1467.75±51.73
28	2-Pentanone	C107879	11.55±1.51	14.97±1.41	8.35±0.37	19.91±2.18	16.07±0.90
	**Acids**						
29	Acetic acid	C64197	7993.55±487.86	5477.58±184.45	4937.66±141.89	4585.35±44.24	4404.78±55.84
30	Propanoic acid	C79094	289.70±18.31	321.59±13.37	326.60±13.05	308.00±12.07	280.01±10.24
	**Pyrroles**						
31	Pyrrolidine	C123751	2425.16±389.57	2249.21±451.54	2456.88±215.88	2297.52±346.97	2380.83±337.40
32	2-Formylpyrrole	C1003298	263.93±44.03	287.18±83.55	266.34±40.02	294.31±73.39	293.33±64.99
	**Hydrocarbons**						
33	n-Propylbenzene	C103651	116.38±10.47	185.99±3.04	97.55±7.78	219.21±6.36	152.79±6.64
	Total		26631.29±434.38	29408.33±831.84	22564.82±526.92	29134.06±1149.55	26733.91±760.00

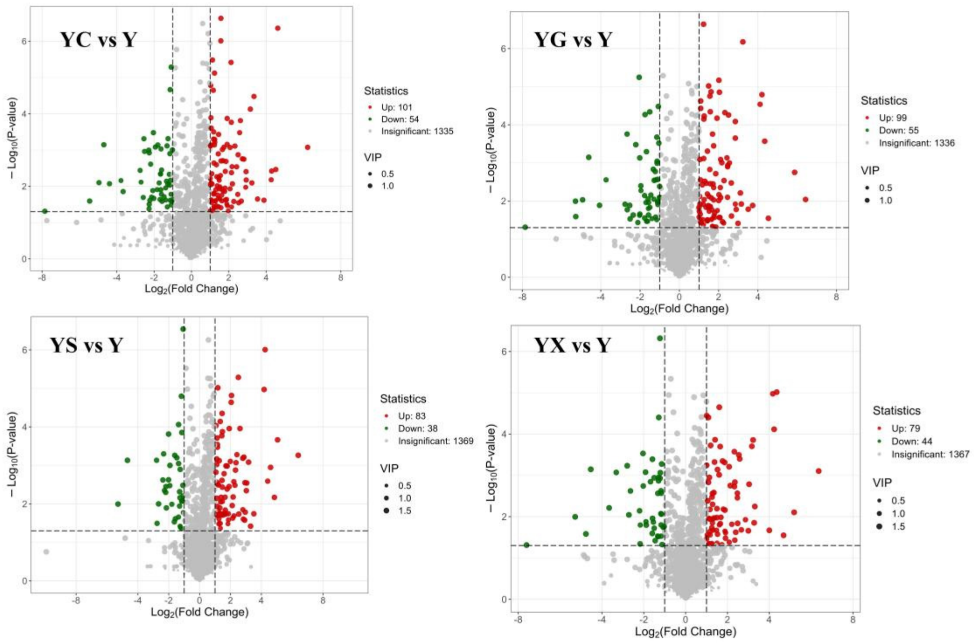

Figure S1. Volcano plot of differential functional metabolites among compared groups (YC vs Y, YG vs Y, YS vs Y, YX vs Y) obtained by UPLC-MS/MS.

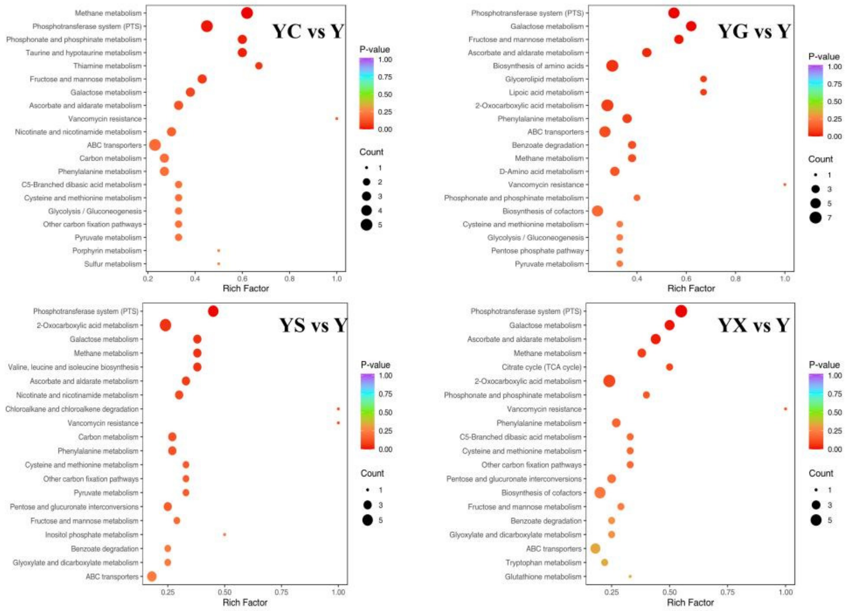

Figure S2. Bubble plots of the top 20 KEGG pathways for differential functional metabolites among compared groups (YC vs Y, YG vs Y, YS vs Y, YX vs Y) obtained by UPLC-MS/MS.

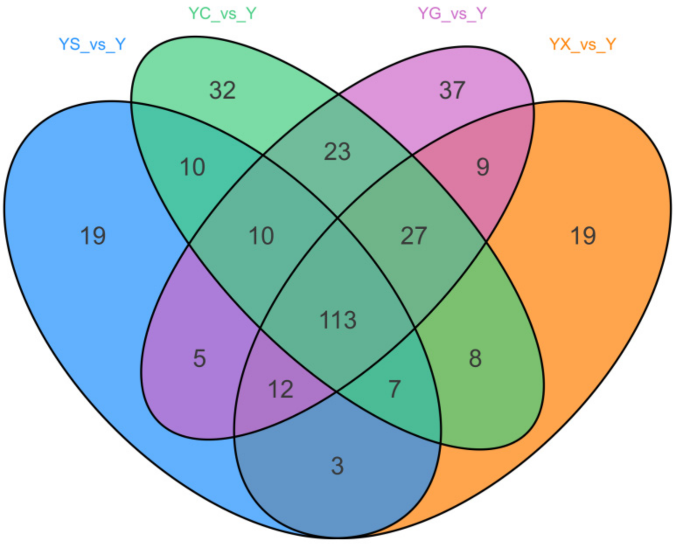

Figure S3. Venn plots of non-volatile metabolites in 5 CPB samples obtained by UPLC-MS/MS, the numbers represent the number of non-volatile compounds in each comparison group, and the overlap represent the number of non-volatile compounds common to multiple groups.

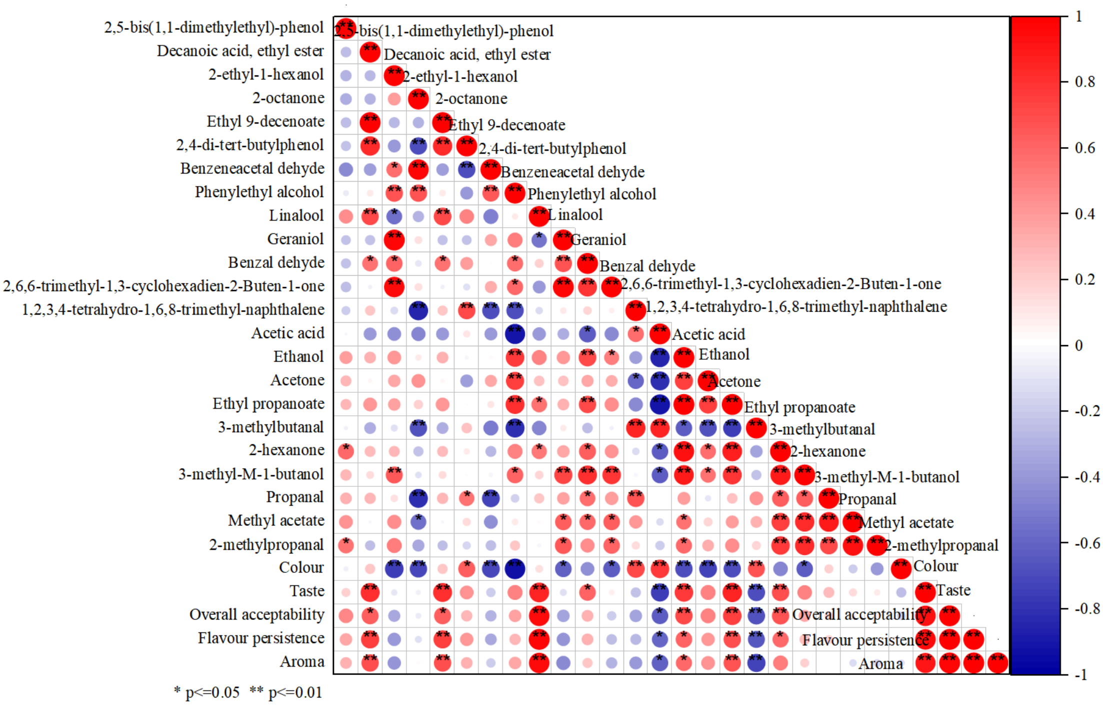

Figure S4. Correlation analysis between sensory evaluation and VOCs in CPB samples fermented by different strains.
